# HIV‐1 viral protein effect on cerebral microvasculature: An in vitro blood–brain barrier model

**DOI:** 10.14814/phy2.70593

**Published:** 2025-09-30

**Authors:** Sinelisiwe Matubatuba, Chontrelle Willemse, Khayelihle Brian Makhathini, Carine Smith, David Fisher, Shireen Mentor

**Affiliations:** ^1^ Division of Cell Biology, Department of Human Biology, Anatomy Building Medical Campus University of Cape Town Cape Town South Africa; ^2^ Neurobiology Research Group, Department of Medical Biosciences, New Life Sciences Building University of the Western Cape Cape Town South Africa; ^3^ Experimental Medicine Group, Department Medicine Stellenbosch University Cape Town South Africa; ^4^ School of Health Professions University of Missouri Columbia Missouri USA; ^5^ Department of Physiological Sciences, Mike De Vries Building Stellenbosch University Stellenbosch South Africa

**Keywords:** blood–brain barrier, human immunodeficiency virus, transendothelial electrical resistance

## Abstract

The central nervous system (CNS) serves as a sanctuary for the Human Immunodeficiency Virus (HIV), which is facilitated by HIV's ability to breach the blood–brain barrier (BBB). BBB dysfunction occurs in the earliest stages of an HIV‐1 infection. The immune‐privileged CNS reduces harmful inflammatory responses, detrimental to the neuronal environment. BBB disruption, however, contributes to comorbidities in HIV, like cerebrovascular disease and neurocognitive problems. A 2‐dimensional in vitro BBB model was employed to assess the effect of HL2/3 cell paracrine factors on select physiological parameters: cell proliferation, viability, toxicity, suppression, and morphology. BBB integrity was assessed using transendothelial electrical resistance measurements. The study utilized immortalized mouse brain endothelial cell monocultures and co‐cultures with the HL2/3 cell line, emulating an in vivo HIV‐1 effect on the BBB. A concentration‐dependent decline in cellular proliferation rates and viability was observed upon exposure to HL2/3 paracrine factors. Moreover, an elevation in cellular suppression, cell death, and cell toxicity was observed. Permeability studies confirmed decreased impermeability after exposure to HIV‐1 viral proteins in select in vitro BBB model systems. The impact of HIV viral proteins on brain capillary endothelium is critical to elucidate pathogen‐induced cerebrovascular disease progression and vascular cognitive impairment in patients.

## 1 INTRODUCTION

HIV (Human Immunodeficiency Virus) remains a major global health challenge, affecting millions of individuals worldwide. According to the World Health Organization, as of 2023, an estimated 39 million people were living with HIV, 25 million of whom are in Africa (World Health Organisation, 2023). While advancements in antiretroviral therapy (ART) have dramatically improved the prognosis for individuals living with HIV, the virus continues to cause complex challenges, particularly concerning its interaction with the central nervous system (CNS) (Osborne et al., 2020). HIV has been found to enter the CNS 4–8 days following peripheral infection; as a result, HIV viral reservoirs may persist in the CNS even before the introduction of ART. HIV‐1 has a ribonucleic acid (RNA) genome, which co‐opts the host machinery to express 9 genes, the most important being group‐specific antigen (gag), envelope (env), gp120, gp41, polymerase (pol); accessory and regulatory proteins, which include: trans‐activator of transcription (tat), regulator of virion (rev), viral infectivity factor (Vif), viral protein U (Vpu), viral protein R (Vpr), and negative factor (Nef) (Gurtler et al., 2016; Li et al., 2015; Zhang et al., 2000).

HIV infection can traverse the brain's barrier systems by infecting immune cells (i.e., leukocytes, lymphocytes, and monocytes) to bridge inflamed brain capillary endothelium. Upon entry into the CNS, HIV infects microglial cells (M1), macrophages, and astrocytes, resulting in downstream effects on the neuronal *milieu*. HIV infections are therefore able to elicit an immune response mediated by cytokines and chemokines, which adversely affect infected host cells concomitant with the release of host inflammatory factors and viral proteins (Chilunda et al., 2019; Gurtler et al., 2016; Martin‐Gayo & Yu, 2019).

With HIV being a neurotrophic virus, it can cause infection of the neural tissue within the CNS and subsequently result in the progression of HIV‐associated neurocognitive disorders (HAND). HAND, if persistent, could result in mild to severe cases of cognitive impairment, despite ART (Heaton et al., 2011; Muñoz‐Moreno et al., 2021).

HAND is found to be prevalent in the later stages of AIDS; despite ART, it is the leading cause of morbidity in up to 50% of individuals living with HIV (Buckley et al., 2021). Moreover, 50% of the HIV‐1‐seropositive patients in the USA are diagnosed with HIV‐associated neurocognitive disorders (Antinori et al., 2007; McArthur et al., 2010). HAND in viraemic individuals is related to ongoing viral infection in the CNS, chronic inflammation, and oxidative stress (Buckley et al., 2021).

The anatomical basis of the neurovascular unit (NVU) is the brain's capillaries, which are comprised of highly specialized and strictly regulated brain endothelial cells (BECs) that serve as a protective interface inhibiting the influx of blood‐borne toxins and pathogens into the CNS, preserving the brain's microenvironment (Osborne et al., 2020). Furthermore, its function as a “stabilizer” of the neuronal *milieu*, BECs, ensuring that the brain is insulated from fluctuations of hormones, harmful substances, and immune factors circulating in the peripheral circulation. BECs thus form an impervious barrier, which ensures the immune privilege (IP) of our brain's microenvironment (Muldoon et al., 2013).

IP can be bypassed in the face of a sufficiently strong immunological response in vascular pathology (i.e., viral infection). In this scenario, privileged brain tissues may be at greater risk of collateral damage due to the increased susceptibility of the natural defenses being breached during vascular inflammation. The literature reports on two existing hypotheses: (i) HIV persists in these sanctuaries during ART and may cause the generation and dissemination of drug‐resistant viruses; (ii) the breakdown of the blood–brain barrier (BBB) contributes to the trafficking of HIV‐1 infected immune cells and subsequent HIV‐1 viral proteins into the brain's microenvironment via the process of extravasation (Atluri et al., 2015; Osborne et al., 2020).

In vitro models of the BBB play a critical role in studying the interactions between HIV‐1 viral proteins and the brain capillary endothelium, providing a controlled and accessible platform to investigate the virus's entry into the CNS. The basis for the BBB lies in the localization and interaction of intercellular tight junction (TJ) protein complexes (i.e., claudin 1/3, − 5, and 12, Zonula occludens (ZO)‐1, 2, 3 and occludin) and junctional adhesion molecules (JAMs) between adjacent BECs (Sasson et al., 2021). Studying HIV‐1 using in vitro BBB models allows for a better understanding of how the viral protein dysregulates BEC barrier properties and its subsequent impacts on the brain's microenvironment. Using established in vitro brain endothelial barrier pathological models allows for the experimental study of these structural and physiological complexities of the BBB.

To date, the lack of knowledge pertaining to the nano‐anatomical development or alteration of barrier formation in the BECs of the BBB during HIV‐1 viral infection continues to persist. Recent work by Mentor and Fisher (2021) investigated the novel morphological ultrastructural development of BECs using high‐resolution electron microscopy. This explorative research study underscores the indiscriminate, detrimental effects of HIV‐1 viral protein‐induced dysfunction (i.e., Tat, Gag, Nef, Env, Rev) on immortalized mouse brain capillary endothelial cells. Permeability, cell proliferation, and the subsequent impedance of direct cell–cell ultrastructural communication during BEC monolayer establishment result in the failure of the BBB to form a restrictive interface in vitro. The study, therefore, further illuminates the ultrastructural dysfunction as a critical developmental step in barrier construction and destruction during health and HIV pathogenesis. To date, no data reporting on how HIV viral proteins affect physiology and pathophysiology have been reported.

The study postulates that the detrimental effect of HIV‐1 proteins on BEC proliferation inevitably compromises the BEC's morphological profile during a viral infection, which alters the structural integrity of an established BBB.

## 2 MATERIALS AND METHODS

### 2.1 Bio‐reagents

Immortalized mouse brain endothelial cells (b.End5, ECACC 96091930, UK) and human papillomavirus‐related endocervical carcinoma cells secreting HIV‐1 paracrine factors (HL2/3, ATCC® ARP‐1294, University Boulevard, Manassas, VA) were cultured in Dulbecco's Modified Eagle Medium‐F12 (DMEM‐F12, Gibco™ No. 10565018, Gaithersburg, MD, USA), supplemented with non‐essential amino acids (NEAA, Gibco™ No. 11140050, Gaithersburg, MD, USA), Penicillin–Streptomycin (Merck No. P4458, Darmstadt, Germany), sodium pyruvate (Merck No. 792500, Darmstadt, Germany), and fetal bovine serum (FBS, Merck, No. 32160409, Darmstadt, Germany). Passage of both cell lines was conducted using Tryple Express Enzyme (1X) (Gibco™ No. 12605010, Gaithersburg, MD, USA) as a gentle cell‐dissociating agent.

### 2.2 Collection of HL2/3 supernatant

The collection of supernatants from HL2/3 cells for the exposure of b.End5 cells to HL2/3‐conditioned media (CM) was carried out through the following protocol: Initially, 1 × 10^6^ HL2/3 cells were seeded in 75 cm^2^ tissue culture flasks (T‐75) and allowed to adhere under standard conditions (37°C and 5% CO_2_) for a duration of 12 h. After the 12‐h incubation period, the media from the flasks were discarded and replenished with fresh media and subjected to an additional 24 h of incubation. Following the 24‐h incubation period, supernatant from the HL2/3 cells was meticulously collected into ice‐cooled 15 mL tubes (LASEC No. T 1943‐1000, Cape Town, SA) and subsequently stored at −80°C for further use.

### 2.3 b.End5 exposure to HL2/3 supernatant

The thawed supernatant collected from HL2/3 cells underwent preparation at varying concentrations using a fresh supplemented DMEM‐12 1:1 nutrient mixture (v/v) to make up the 20% and/or 25%, 40% and 75% HL2/3‐CM. The 100% represented an undiluted HL2/3 supernatant. This would demonstrate the effect of increasing viral load on the physiology of b.End5 cells. Depending on the specific assay requirements, b.End5 cells were seeded at different densities and subsequently incubated at 37°C with 5% CO_2_ for a period of 12 h to 18 h to facilitate cellular adherence.

Following successful cell attachment and dependent on the experimental design, b.End5 cells were subjected to chronic exposure to HL2/3‐CM at concentrations of 20%, 25%, 40%, 75%, and 100%. All assays were conducted in triplicate (*n* = 3) and repeated thrice for repeatability over specified time intervals of 24, 48, 72, and 96 h. To maintain consistent conditions, media replenishment occurred daily, ensuring that b.End5 cells received sufficient substrates for normal metabolic functioning and were consistently exposed to the predetermined concentration of HL2/3‐CM throughout each designated time point.

### 2.4 Study design

The study was based on an established 2‐D HIV blood–brain barrier (BBB) (HL2/3 paracrine secretions exposed to b.End5 cells) model. Experimentation was either non‐sequential (Cell Viability and Cell Toxicity Assay) or sequential (Transendothelial Electrical Resistance (TEER) and High‐Resolution Scanning Electron Microscopy). All b.End5 cultures received daily freshly made HL2/3‐CM from 24 to 96 h. This ensured maintenance of consistent conditions and that the cultures received sufficient substrates for normal metabolic functioning. The non‐sequential experiments received HL2/3‐CM at concentrations of 20% and/or 25%, 40%, and 75%, and different exposed cultures were harvested every 24 h. Whereas the sequential experiments received HL2/3‐CM at concentrations of 20%, 45%, 75%, and 100%, and the same experimental cultures were used until experimental termination. All assays were conducted in triplicate (*n* = 3) and repeated thrice for repeatability over specified time intervals of 24, 48, 72, and 96 h.

### 2.5 Cell viability and cell toxicity assay

The trypan blue assay was employed to quantitatively assess physiological parameters, encompassing cell viability, toxicity, and cellular proliferation. The b.End5 cells were seeded onto 12‐well plates (Thermo Scientific™, No. 150628, Johannesburg, SA) at a density of 2.5 × 10^3^ cells/well and incubated for 12 h to facilitate attachment. Subsequently, b.End5 cells were exposed to 20%, 40%, or 75% concentrations of HL2/3‐CM for durations ranging from 24 to 96 h. This experiment was conducted thrice, with three technical repeats (*n* = 3). A trypan blue exclusion assay was conducted by pipetting a total of 10 μL of b.End5 cells into Eppendorf tubes (epi, Lasec No. Z657506, Cape Town, SA), and 10 μL of 0.4% trypan blue (Sigma‐Aldrich No. 93595, Darmstadt, Germany) was added, creating a 1:1 ratio (i.e., 10 μL cell suspension: 10 μL trypan blue), and cell counting was conducted using an inverted‐phase light microscope. Dead cells, permeable to trypan blue, appeared blue, whereas live, viable cells, with intact membranes impermeable to trypan blue, were identified.

### 2.6 XTT assay

The XTT (2,3‐bis‐(2‐methoxy‐4‐nitro‐5‐sulfophenyl)‐2H‐tetrazolium‐5‐carboxanilide) assay is a colorimetric assay and was used to assess the indiscriminate effects of HIV‐1 viral proteins on mitochondrial dehydrogenase activity on brain endothelial cells as a proxy for cell viability. The b.End5 cells (5 × 10^4^ cell/well) were seeded in a 24‐well plate and treated at 25%, 40%, 75%, and 100% HL2/3‐CM from 24 to 96 h using mitochondrial activity as a proxy (American Type Culture Collection, 2011). At each time point, cells were trypsinised using Tryple, reseeded at 5 × 10^3^ cells/well in 100 μL of complete medium in a 96‐well plate, and allowed to attach for 12 h in an incubator at standard tissue culture conditions (i.e., 37°C and 5% CO_2_). Thereafter, 50 μL of reconstituted XTT reagent was added to each well, the plate was wrapped in aluminium foil to protect it from light, and incubated under standard tissue culture conditions for 4 h. After incubation, absorbance was measured at 450 nm using an ELISA plate reader.

### 2.7 2‐D experimental model‐bicameral system

**FIGURE 1:**
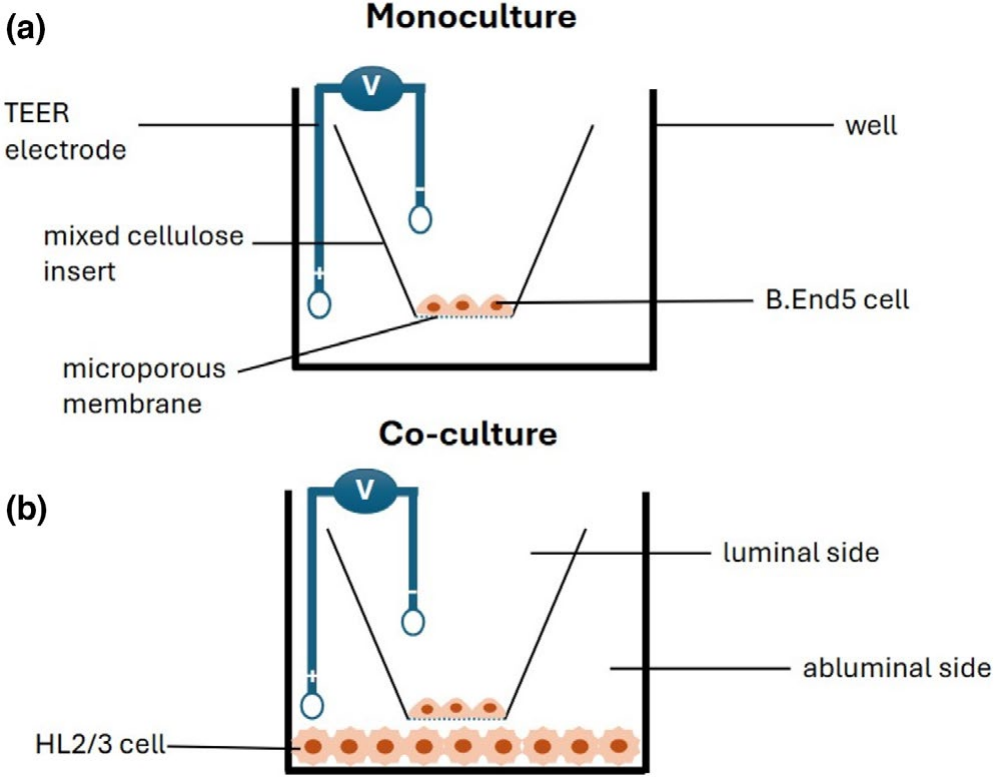
The bicameral system emulating the blood–brain barrier in vitro (a) The b.End5 cells were cultivated on mixed‐cellulose inserts and exposed to HL2/3‐CM luminal to the monoculture, simulating HIV infection in the blood. (b) In the co‐culture, b.End5 cells were cultivated on the mixed‐cellulose inserts and exposed to HIV‐1 proteins secreted by the HL2/3 cells seeded abluminal to the insert, simulating CNS infection in vivo. Transendothelial electrical resistance (TEER) was measured across the b.End5 monolayers under conditions that mimic the luminal and abluminal aspects of the BBB in the context of an HIV‐1 infection. TEER detects the movement of ions across cells, and this can be either transcellular or paracellular movement.

A bicameral system was employed for the TEER experiment in the development of this in vitro BBB model. In this setup, the apical compartment held media in the inserts, which contained a seeding density of 5 × 10^4^ cells/ insert/well in 300 μL, within 24‐well plates (Merck No. 32190102, Darmstadt, Germany) for a duration ranging from 24 to 216 h. The b.End5 monolayers within the insert serve as a representation of the blood (i.e., luminal side) in vivo. Concurrently, the basolateral compartment of the well contained 500 μL media representing the brain (i.e., abluminal side) in vivo, mimicking the brain capillary endothelium bed (Figure 1a). The co‐culture system in Figure 1b introduces HL2/3 cells into the abluminal compartment, mimicking a CNS infection (Figure 1b). These experimental configurations allowed for the investigation of TEER across the b.End5 monolayers, mimicking the physiological barriers encountered at the blood–brain interface (Figure 1).

### 2.8 Transendothelial electrical resistance (TEER)

TEER, a testing method employed for the quantification of in vitro BBB monolayer permeability, was utilized in this study to assess the impact of HIV‐1 proteins on b.End5 monolayer permeability. An in vitro BBB was established by seeding b.End5 cells onto microporous, mixed‐cellulose, Millicell cell culture insert membranes (Merck No. 32011202, Darmstadt, Germany) with a pore size of 0.45 μm, a filtration diameter of 12 mm, and an effective filtration area of 0.6cm^2^. These inserts were placed in 24‐well plates, in triplicate (including 3 technical blank inserts, without cells). TEER was measured daily from 24 to 216 h using the Millicell®‐ERS Voltohmmeter (American Laboratory Trading No. 25439, San Francisco, USA).

### 2.9 Effect of HL2/3‐conditioned media on b.End5 cell monolayer

A density of 5 × 10^4^, b.End5 cells/insert/well were seeded onto mixed‐cellulose inserts within a 24‐well plate for an experimental period of 120 h; cells were cultivated in normal conditions and with daily replenishment of medium. At 120 h, upon reaching optimal TEER readings, b.End5 cells were exposed to select concentrations (20%, 40%, 75%, or 100%) of HL2/3‐CM via the luminal compartment of the bicameral system. The cells were seeded on the same plate, and TEER was recorded sequentially at select time points on the same set of wells. The b.End5 cells were subject to daily exposure to the above‐mentioned concentrations from 120 h to 216 h. Resistance readings were taken thrice in triplicate/day (*n* = 3) from 24 to 216 h.

### 2.10 Co‐culture of b.End5 cells and HL2/3 cells

A density of 5 × 10^4^ b.End5 cells/insert/well was seeded onto mixed cellulose inserts, cultured within a 24‐well plate for 120 h under normal conditions with daily replenishment of media. For HL2/3 cell seeding, 5 × 10^4^ HL2/3 cells were seeded at a volume of 1 mL into 12‐well plates after 72 h. Spent media was discarded after 24 h of incubation. HL2/3 cells were allowed to grow for an additional 24 h. The co‐culture was initiated after 120 h; the b.End5 inserts grown in the 24‐well plate were transferred to the 12‐well plate containing HL2/3 cells. The co‐culture system was independently investigated and comparatively analyzed with the 100% HL2/3‐CM in the monoculture model (Figure 6) to distinguish between the effects of luminal and abluminal exposure to HL2/3‐CM on in vitro BBB dysfunction. TEER was measured thrice, in triplicate/day (*n* = 3) from 24 to 216 h.

### 2.11 High‐resolution scanning electron microscopy

High‐resolution scanning electron microscopy (HRSEM) is an imaging technique that allows for the analysis of monolayer development in healthy and diseased states on a nanoscale. BECs (b.End5 cells) were grown at 37°C, at 5% CO_2_ on Millicell filter inserts at 5 × 10^4^ cells/insert/well. Upon cellular confluence, monolayers in the TEER studies were exposed to HL2/3‐CM and subsequently fixed with 2.5% glutaraldehyde solution (Faso et al., 1994). The biological sample was dehydrated in a graded series of ethanol concentrations and critically dried using samples sputter coated with gold and imaged using the Tescan MIRA SEM high‐resolution field‐emission gun SEM (Mentor et al., 2022). All images were captured using an in‐lens secondary electron detector.

### 2.12 Statistical analysis

Statistical analysis was conducted using GraphPad Prism version 10.0.2 (GraphPad Software, San Diego, CA, USA). The data were presented as mean ± SEM. The Shapiro‐Wilk test was utilized to test for normality. To assess statistical significance between different groups, one‐way ANOVA and Kruskal–Wallis tests were employed for independent experimental assays, and two‐way repeated measures ANOVA or two‐way mixed ANOVA was employed for dependent experiments. Post hoc tests, specifically, Tukey's, Dunnett's, and Dunn's, were applied for multiple comparisons in parametric and non‐parametric data, respectively. Significance levels were denoted as follows: *p < 0.05, **p < 0.01, ***p < 0.001, ****p < 0.0001. These designations indicated the degree of statistical significance for the observed results.

## 3 RESULTS

### 3.1 HIV‐1 viral protein effect on brain endothelial cellular proliferation

**FIGURE 2:**
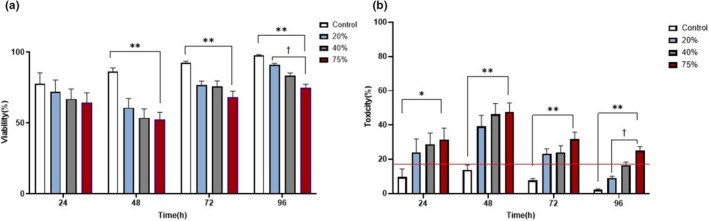
The effects of daily exposure to HL2/3‐conditioned media (CM) (20%, 40%, and 75%) on b.End5% Viability and % Toxicity at 24, 48, 72, and 96 h. (a and b), depicts the effects of HIV‐1 viral proteins on % b.End5 viability and toxicity, from 24 to 96 h. A red solid line is denoted across the timeline for b.End5 cell toxicity and this line represents the maximum toxicity for the control (b). Data are expressed as mean ± SEM (*n* = 3). Statistical significance was determined with one‐way ANOVA and Tukey and Dunnette's post hoc test for multiple comparisons. *p < 0.05, **p < 0.01, †*p* < 0.001 denotes the statistical significance.

Figure 2 a–b depicts b.End5 cells exposed to select concentrations of HL2/3‐CM, and percentage viability and toxicity were calculated daily. b.End5 cell viability and toxicity were investigated to ascertain the effects of HIV‐1 viral protein on an in vitro HIV‐BBB model. A concentration‐related decrease in % b.End5 cell viability was observed from 24 to 96 h post‐exposure (Figure 2a). From 48 to 96 h after exposure, a significant difference (*p* < 0.01) was noted in the viability of the control and all select concentrations. Additionally, at 96 h, a statistically significant decrease (*p* < 0.001) in b.End5 cell viability was noted between b.End5 cells exposed to 20% and 75% of HL2/3‐CM. b.End5 cell toxicity displayed an inverse trend to cell viability; from 24 to 96 h post‐exposure, a concentration‐related increase in % cell toxicity was observed (Figure 2b). At 24 h post‐exposure, cell toxicity of the control was significantly (*p* < 0.05) different from b.End5 cells exposed to 75% of HL2/3‐CM. From 48 to 96 h post‐exposure, b.End5 toxicity of the control was significantly different from b.End5 cell toxicity in all experimental groups (*p* < 0.01). Additionally, a statistically significant increase (*p* < 0.001) in cell toxicity was noted between b.End5 cells exposed to 20% and 75% of HL2/3‐CM. The % toxicity levels of the control groups were significantly different from the experimental groups, always observed below 20% as indicated by the red solid line (Figure 2b).

### 3.2 HIV‐1 viral protein impact on brain endothelial cell suppression

**FIGURE 3:**
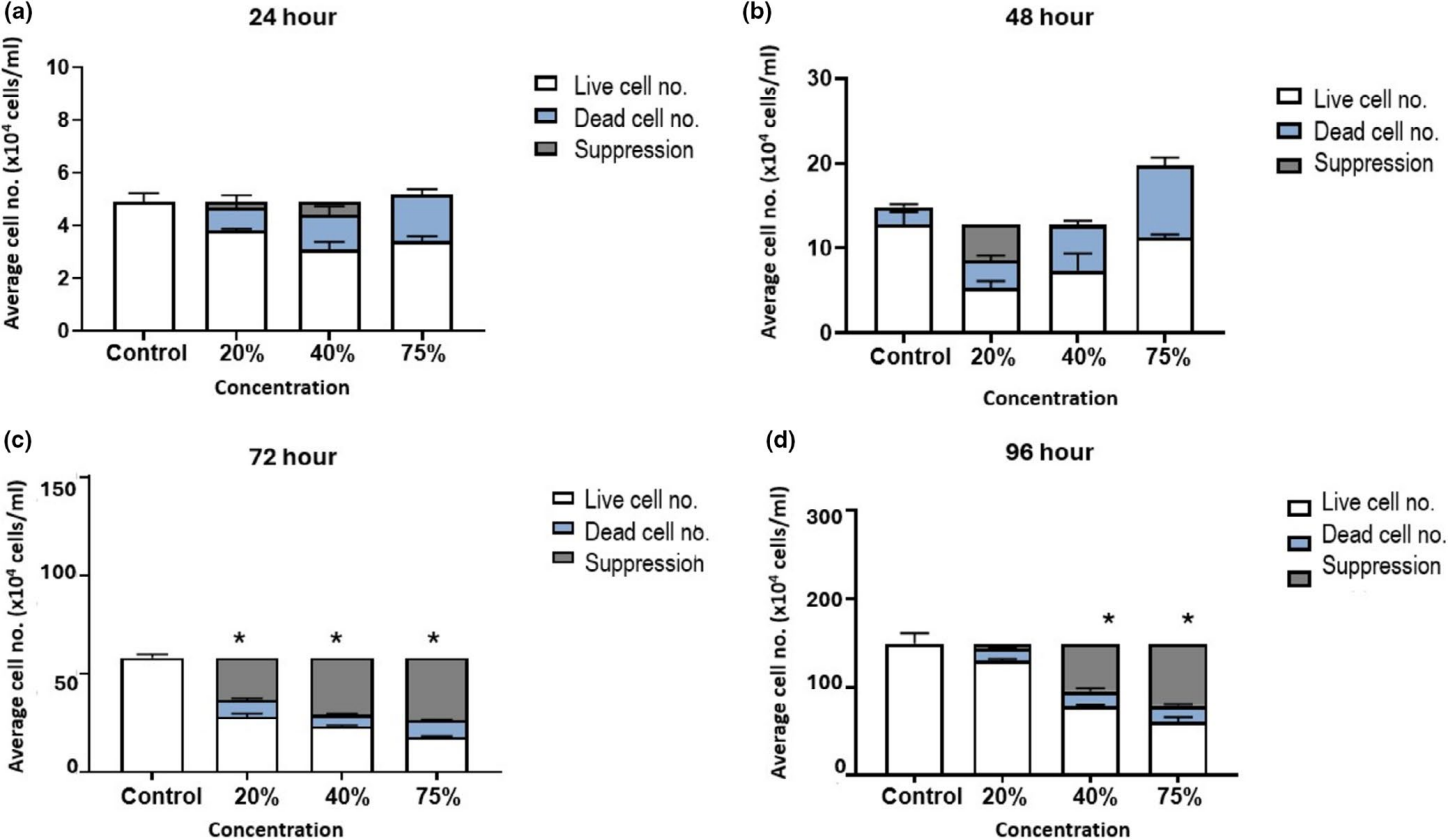
The effect of daily exposure of HL2/3‐CM on b.End5 cell division. b.End5 cells were exposed to 20%, 40%, and 75% concentrations of HL2/3‐CM for (a) 24 h, (b) 48 h, (c) 72 h, and (d) 96 h. The white bars illustrate the live cell numbers, and the blue bars illustrate the dead cell numbers. The gray bars refer to the difference between control cell numbers and the sum of treated cells and their dead cell numbers. Data are expressed as mean ± SEM (*n* = 3).

Daily exposure to HL2/3‐CM on b.End5 cellular proliferation was assessed from 24 to 96 h to determine the HIV‐viral protein impact on the rate of BEC division (Refer to white bars). Figure 3 depicts the effects of HIV‐1 proteins on b.End5 cellular proliferation over a 96‐h period. At 24‐48 h post‐exposure, no significant difference was noted between the control and the selected concentrations of HL2/3 supernatant (Figure 3a,b). At 72 h after exposure, live b.End5 cell numbers that were exposed to HIV‐1 proteins (20%, 40%, and 75% of HL2/3‐CM) showed statistically significant decreases in live cell numbers (*p* < 0.05) compared to control samples (Figure 3c). At 96 h post‐exposure, a statistically significant, concentration‐related decrease in b.End5 live cell numbers was noted at 40% and 75% of HL2/3 CM exposure compared to the controls (*p* < 0.05, respectively) (Figure 3d).

A concentration‐related rise in b.End5 dead cell numbers was noted 24 h post‐exposure (Figure 3a). At 48 h post‐exposure, an increase in b.End5 dead cell numbers was noted between the control and the 40% and 75% of HL2/3‐CM conditions. At 72 h after exposure, statistical significance (*p* < 0.05) was seen between the control and b.End5 cells exposed to 20%–75% of HL2/3‐CM. A trend of increased dead cells was noted throughout the 96 h experimental timeframe, with a significant difference (*p* < 0.0001) present between the control and all select concentrations. The differences between levels of live cells in both the control and experimental conditions were not fully attributable to dead cell numbers post‐exposure. The observed differences, therefore, had to be attributed to the suppression of cell division.

### 3.3 HIV‐1 viral protein effect on mitochondrial activity

**FIGURE 4:**
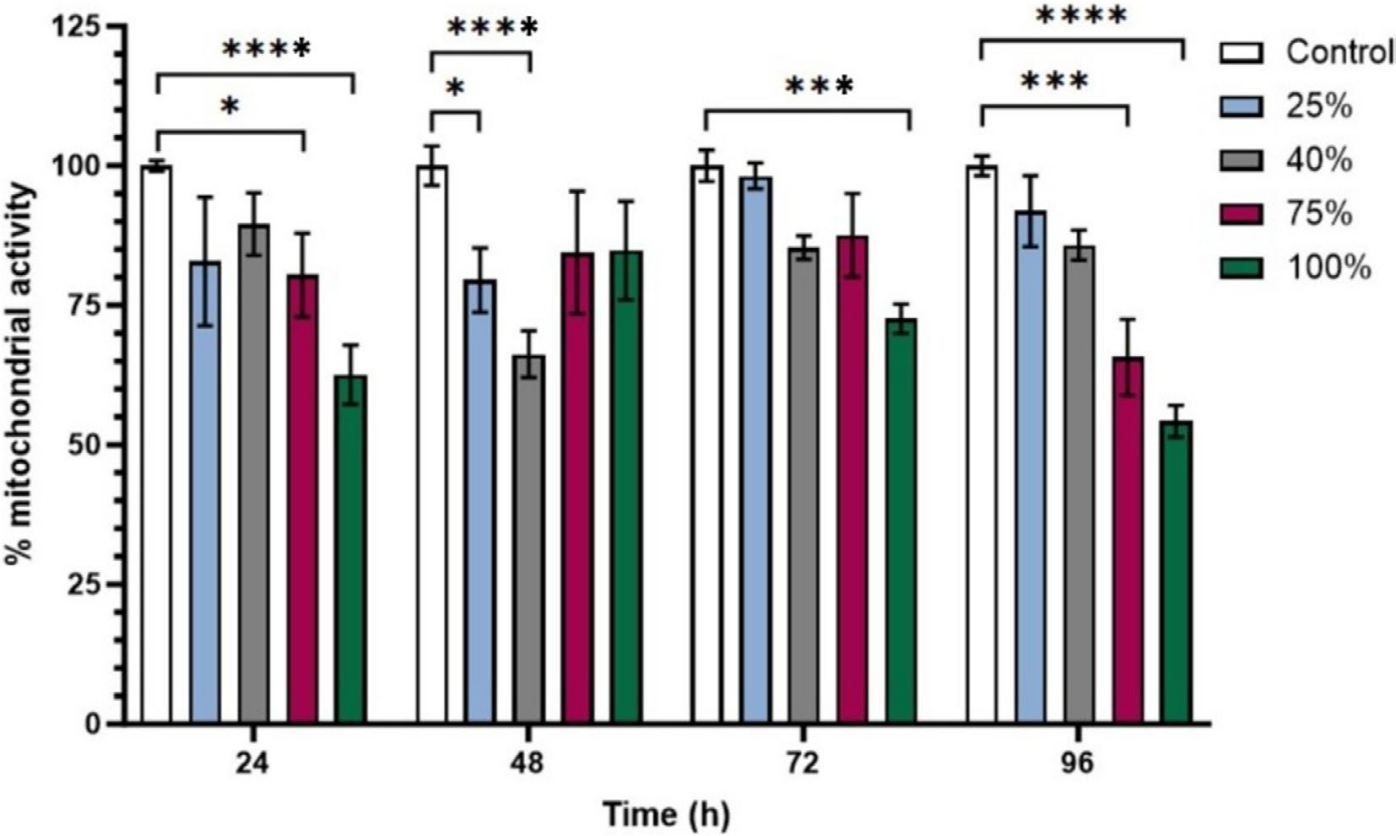
The effect of daily exposure to HL2/3‐CM on percent mitochondrial activity of b.End5 monolayers. Monolayers were chronically (i.e., daily) exposed to 25%, 40%, 75%, and 100% concentrations of HL2/3‐CM for 24, 48, 72, and 96 h. Data are represented as mean ± SEM (*n* = 3). Statistical significance was determined using Kruskal‐Wallis and Dunn's post hoc test and is denoted by the following: *p < 0.05, ***p < 0.001, and ****p < 0.0001.

In Figure 4, at the 24‐h time point, monolayers treated with 25%, 40%, and 75% HL2/3 supernatant showed a statistically significant difference in mitochondrial activity at 75% and 100% post‐exposure, compared to the controls (*p* < 0.0002). At 48 h, significant differences were observed for the monolayers treated with 25% and 40% supernatant, which showed a significant decrease in mitochondrial activity compared to the controls (*p* < 0.0005). The 72‐h exposure exhibited a trend like the 24‐h exposure, with the monolayers treated with supernatant showing a significant decrease in mitochondrial activity at 75% and 100% HL2/3 exposure compared to controls (*p* < 0.003). The 96‐h time point reveals a clear concentration‐dependent reduction in mitochondrial activity, with the 75% and 100% groups showing significant decreases in mitochondrial activity compared to the controls (*p* < 0.001, respectively) (Figure 4).

### 3.4 Transendothelial electrical resistance (TEER)

**FIGURE 5:**
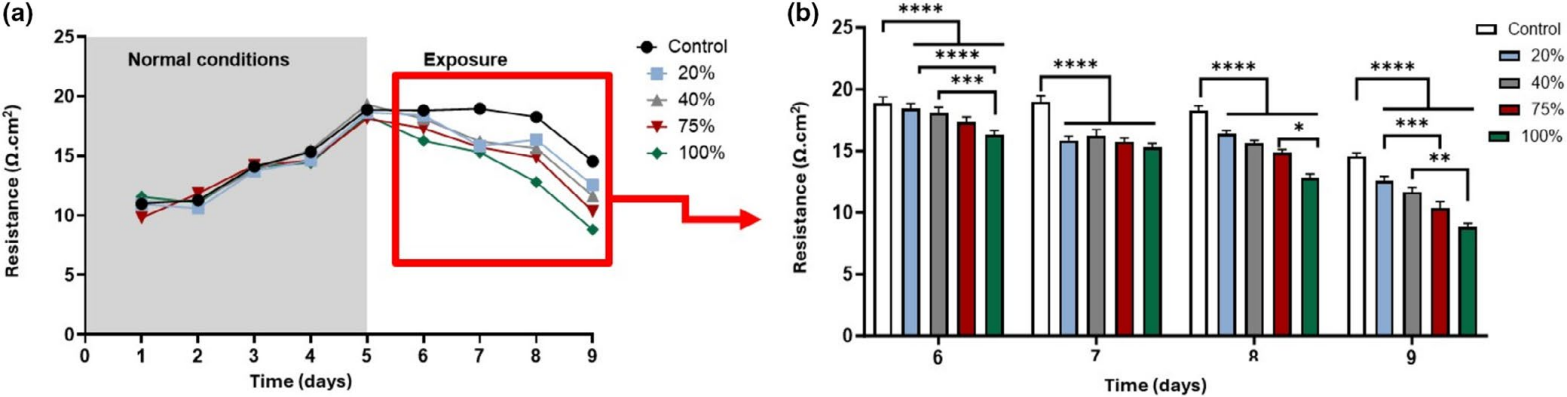
The effect of HIV‐1 proteins on b.End5 monolayer permeability. (a) shows resistance readings across the entire experimental timeline; (b) shows the resistance readings during the days of exposure only, using a histogram which allows for statistical representation of the data. Data expressed as mean ± SEM (*n* = 3). Statistical significance was determined with two‐way repeated measures ANOVA, and for multiple comparisons, post hoc Tukey tests were performed. *p < 0.05, **p < 0.01, ***p < 0.001, ****p < 0.0001 designates the statistical significance.

The effect of HL2/3‐CM on the integrity of an established in vitro BBB was analyzed employing TEER measurements to assess in vitro BBB permeability post‐exposure to HIV‐1 viral proteins. Treatment was introduced once TEER plateaued (Figure 5a), which indicated that a confluent monolayer had been established. A significant decrease (*p* < 0.0001) in resistance is seen between the control and all the select concentration groups from Day 6 to 9 (i.e., 4 days post‐exposure) (Figure 5a,b). At Day 6, 100% of HL2/3 supernatants was significantly decreased compared to the lower concentrations (*p* < 0.006). From Day 8 to 9 (i.e., 72 and 96 h post‐exposure), significant decreases in TEER were observed at the 20% exposed b.End5 groups compared to the higher concentration of 75% HL2/3 supernatant (*p* = 0.002), and a significant decrease in TEER was seen at 40% compared to 100% (*p* = 0.02).

**FIGURE 6:**
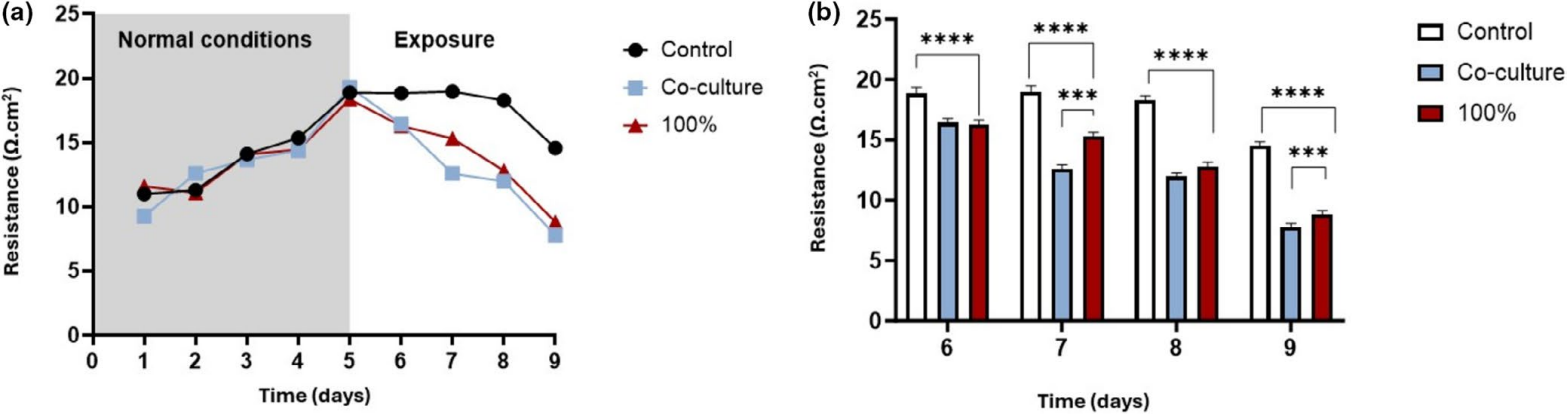
Changes in b.End5 monolayer permeability after exposure to 100% HL2/3‐CM and in co‐culture with HL2/3 cells. (a) displays trends in permeability across the entire experimental timeline while (b) shows the changes in TEER following 100% exposure and co‐culture. Data expressed as mean ± SEM (*n* = 3). Statistical significance was determined with two‐way mixed ANOVA and Tukey's and Dunnette's post hoc tests for multiple comparisons between control and experimental samples. ***p < 0.001 designates statistical significance between experimental groups and ****p < 0.0001 designates the statistical significance differences between control and experimental samples.

The impact of 100% HL2/3‐CM and an HL2/3 co‐culture on a b.End5 cell monolayer was investigated as an independent repetition of the study conducted in Figure 6, in parallel with the co‐culture system to distinguish between the effects of luminal and abluminal exposure to HL2/3‐CM on in vitro BBB dysfunction. Figure 6a illustrates that, prior to exposure, there was minimal disparity in TEER among the three groups. After exposure, a notable reduction in TEER is evident from 144 h (Day 6) to 216 h (Day 9) (i.e., 24–96 h post‐exposure) between the control group and the b.End5 cells exposed to 100% HL2/3‐CM, as well as the co‐culture with HL2/3 cells (*p* < 0.0001) (Figure 6a,b). Moreover, a significant divergence in TEER is observed between b.End5 cells in co‐culture and b.End5 cells exposed to 100% HL2/3‐CM at 168 and 216 h (*p* < 0.001).

### 3.5 Brain endothelial cellular morphology

**FIGURE 7:**
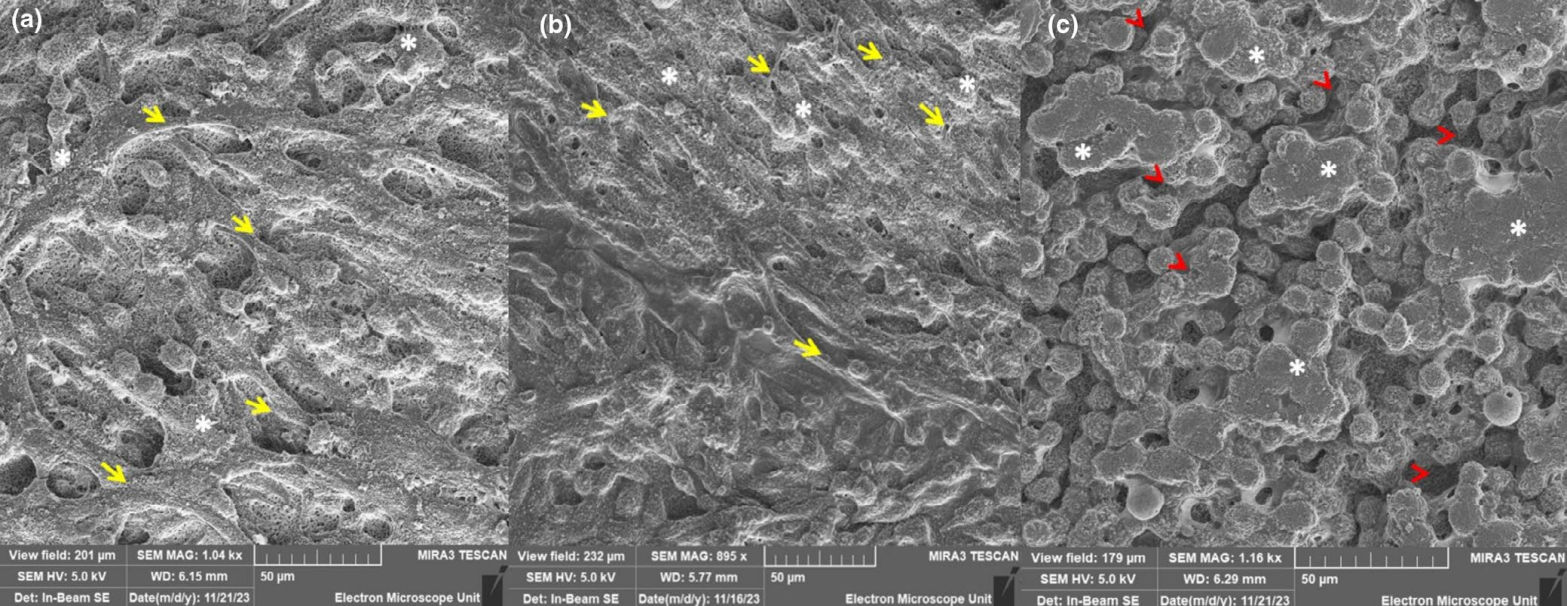
The comparative analysis of monoculture and co‐culture systems of an HIV‐1 in vitro BBB model. (a) The SEM micrograph displays monolayer establishment within a monoculture system, where BECs were exposed to standard supplemented tissue culture medium, devoid of HIV‐1 viral proteins (Control), showing continuous cell–cell connections between BECs growing in proximity, see yellow arrows in (a); (b) The SEM Micrograph shows monolayer establishment within a monoculture bicameral system, where BECs were apically (i.e., within the insert) exposed to 100% HL2/3 CM (i.e., HIV‐1 viral proteins); (c) This SEM micrograph shows BEC monolayer disestablishment when co‐cultured with HL2/3 cells within the basal compartment (i.e., within the well), which mimics a CNS HIV‐1 infection and underscores the detrimental effect of HIV viral proteins on endothelial barrier establishment, showing discontinuity between BECs. The short yellow arrows underscore areas of direct cell–cell communication through nanotube formation and the red arrowheads indicate the absence of nanotube formation. The *asterisks indicates the cell membrane surfaces in (a and b) The formation of nanovesicles accruing on the BEC membrane and in (c) The BEC membrane devoid of nanovesicles (Scale bar = 50 μm).

The BEC monolayer established within the monoculture BBB model illustrates the observable formation of a “lawn” of BECs forming a continuous, confluent monolayer. Extensive cell–cell interaction displays convoluted paracellular spaces and the ultimate sealing of paracellular shunts. Moreover, observable nanovesicles were amassing on the BEC membrane surface (Figure 7a,b) (Refer to * asterisks). Conversely, little to no nanovesicles are seen amassing on the BEC surface in the co‐culture system, despite cell confluence. The lack of nanovesicles (Refer to asterisks) results in the subsequent failed generation of tunneling nanotube (TUNT) formation (Figure 7c), which appears to extend between adjacent BECs as seen in Figure 7a,b (Refer to short yellow arrowheads). Furthermore, the lack of TUNTs and cytoplasmic projections seen in Figure 7c (Refer to red arrowheads) results in a “broken”/discontinuous cell monolayer between adjacent BECs grown on the mixed cellulose insert (Refer to red arrowheads).

**FIGURE 8:**
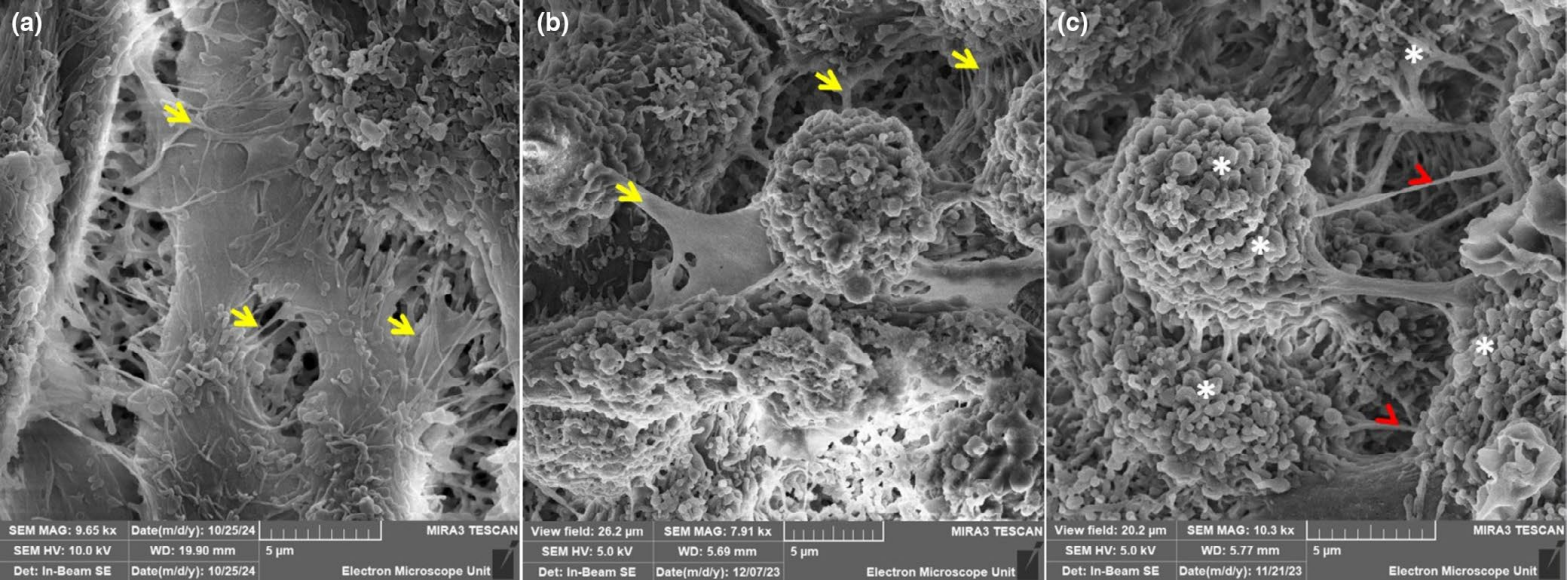
The comparative analysis of morphological variations within monoculture and co‐culture systems of an HIV‐1 in vitro BBB model. a) Represent nanotubule formation within a monoculture, where BECs were exposed to standard supplemented tissue culture medium, apically, devoid of HIV‐1 viral proteins and exhibiting tunneling and tethering nanotubules (TUNTs and TENTs), See yellow arrows in (a); (b) Represents nanotubule formation within a monoculture bicameral system, where BECs were exposed to 100% HL2/3‐CM (i.e., HIV‐1 viral proteins) apically or within the insert, exhibiting a degree of ultrastructural development in the form of tethering and tunneling nanotubules (See short yellow arrows in (b); (c) Represents BEC nanotubule malformation when co‐cultured with HL2/3 cells within the basal compartment, an experimental system, which mimics a CNS HIV‐1 infection and underscores the detrimental effect of HIV viral proteins on endothelial barrier establishment, showing a clear breakdown of the paracellular seal (See *asterisks and red arrowheads in (c), Scale bar = 5 μm).

The technical platform provided by high‐resolution scanning electron microscopy (HRSEM) illuminated the nano‐structural alteration incurred by HIV‐1 protein‐induced dysregulation of immortalized mouse BEC monolayers as the nano‐anatomical basis for BBB integrity. HRSEM provides high‐resolution micrographs that clearly depict the morphological topography of the b.End5 confluent monolayer. Micrographs show how the paracellular spaces are sealed and the role tethering and tunneling nanotubes (TENTs and TUNTs, respectively) play in this process (for a review of these morphological structures), see (Mentor & Fisher, 2021) (Figure 8a,b). The BEC nanotubule generation within the monoculture BBB model illustrates observable cell–cell interaction, with prolific formation of nano‐ultrastructures between the BECs localized in proximity (Refer to short yellow arrows in Figures 7a,b and 8a,b). Little to no TUNTs and TENTs appear to extend between adjacent BECs, as seen in Figures 7c and 8c. Furthermore, the monolayer in Figure 7c appears compromised, especially within the paracellular domains, with no observed nanotubule (NT) formation and complete breakdown of any cytoplasmic extensions between adjacent BECs grown on the mixed cellulose insert (Refer to red arrowheads). Furthermore, much of the mixed cellulose esters insert, which serves as the basal substrate, is exposed (Refer to *asterisks), which displays failed juxtapositioning of adjacent BEC membranes, growing in proximity and thus no subsequent hemifusion of BEC apicolateral membranes.

## 4 DISCUSSION

Investigating the effects of HIV‐1 viral proteins on the in vitro BBB aims to replicate cerebral microvascular dysfunction within an HIV‐diseased state in vivo. The experimental modality employed in this research investigation allowed for the study of HIV‐1 viral protein interactions with the brain capillary endothelial cells under controlled conditions. The BBB forms the protective interface between viraemic infection and is the frontline of defense against CNS infection (Vastag et al., 2022; Wang et al., 2020). In the case of cerebral vascular disease or HIV‐induced inflammation, viruses can traverse the brain's endothelial barrier interface and infect the CNS.

Alterations in BBB integrity occur in the earliest stages of this viral infection (Li et al., 2015; Peluso et al., 2013; Wright et al., 2015) and persist throughout the infection. Given that the BBB is crucial to maintaining the brain's homeostatic environment, it is not unexpected that HIV‐associated neurocognitive disorders (HAND) are associated with acute HIV‐1 infection (Heaton et al., 2011). Given that HIV reservoirs are present in HIV‐infected people and that they are active, as implicated by HAND, we investigated the effects of HIV‐derived proteins secreted by HL2/3 cells on BECs as well as their permeability effects on b.End5 cell monolayers.

### 4.1 Effect of HIV‐1 viral proteins on cellular proliferation

This research study investigated the indiscriminate effect of HIV‐1 viral proteins on select, crucial physiological parameters. Brain endothelial cellular (BEC) proliferation is influenced by two main co‐parameters, toxicity and rate of cell division. The proliferation experiments showed that chronic exposure to viral proteins decreased b.End5 cell numbers and increased the number of dead cells in a concentration‐related manner (see Figures 2a,b and 3a,b). Many studies report on cell proliferation in terms of viability (%) and toxicity (%), both of which reflect the “blunted” data of live and dead cell numbers. Although we report viability and toxicity data for the sake of comparison with previously published data, it is difficult to analyze rates of cell division using the parameters of viability and toxicity.

Therefore, using live cell and dead cell analysis (Figure 3), we show that dead cell numbers increased upon treatment with CM, which illustrates that HIV‐derived proteins cause a low level of increased toxicity of BECs; the dead cells, however, cannot solely account for the decrease in live cells. It can, therefore, be deduced that the decrease in live cell numbers must be due to a suppression in the rate of cell division. This demonstrated the effects of increasing viral load and alludes to the repercussions of chronic exposure to HIV‐1 viral proteins on the brain's capillary endothelium.

Although these findings are supported by the literature, which endorses the fact that an increased viral load augments cytotoxic effects of HIV‐1 infection (Green et al., 2005; Guha et al., 2016; Marincowitz et al., 2019; Kallianpur et al., 2020; Weber & Weiss, 1988) (Figures 2 and 3), this is the first time that we show the effects of HIV‐derived proteins on the endothelial cells of brain capillaries. Furthermore, it is well reported that HIV‐1 proteins can trigger apoptosis or necrosis in other cell types leading to rapid cell death (Anand et al., 2018; Osborne et al., 2020; Strazza et al., 2011). Moreover, the trans‐activator of transcription (Tat) viral protein has been implicated in triggering endothelial cell apoptosis by activating endoplasmic reticulum stress and mitochondrial dysfunction, leading to cell death (King et al., 2006; Ma et al., 2016; Marino et al., 2020). HIV‐1 viral protein R (Vpr) and envelope protein glycoprotein 120 (Gp120) induce the expression of matrix metalloproteinases (MMPs), which function in degrading the extracellular matrices and the basement membrane, resulting in cell death (Anand et al., 2018; Guha et al., 2012; Louboutin & Strayer, 2012), all of which endorse our findings of HIV‐derived proteins causing increased toxicity. Furthermore, the findings on daily exposure to HL2/3 supernatant further support the observed viral protein‐induced suppression in live cell numbers. For the first time, our findings show that daily exposure to elevating levels of HIV‐derived protein results in decreased cell numbers, which we hypothesize is caused by a suppression of brain capillary endothelial cell division. This notion is endorsed by literature reports on HIV‐1 Vpr in other cell types having the propensity to induce cell cycle arrest by promoting the expression of proteosomes, namely ubiquitin, which amounts to degradation of cell cycle regulators of phosphorylation through active inhibition of cyclin‐dependent kinase 1 (cdk1) inhibiting the cell's progression to the mitotic growth phase (Rogel et al., 1995; Zhao & Elder, 2005).

Furthermore, the data showing the effects of HIV‐1 viral protein on cell proliferation is endorsed by the effects of HL2/3 paracrine factor on b.End5 % mitochondrial reductase capacity, which underscores the detrimental effect of HIV‐1 viral proteins on b.End5 cellular energetics.

### 4.2 HIV‐1 viral protein effect on mitochondrial activity

The XTT results presented in Figure 4 indicate that HIV‐1 viral proteins significantly affect mitochondrial activity and the viability of BECs. Monolayers treated with 25% HL2/3‐CM maintained mitochondrial activity comparable to controls throughout the 96‐h period. In contrast, exposure to 75% and 100% supernatants led to a marked, concentration‐dependent decline in mitochondrial activity, with a statistically significant reduction observed from as early as 24 h (*p* < 0.0002). At 48 h, the impact of viral protein exposure was minimal at lower concentrations (25%, 40%), yet still showed significant differences compared to controls. However, the 100% supernatant caused a sustained and pronounced decrease in mitochondrial activity between 24 and 96 h (*p* < 0.05). These findings suggest that higher concentrations of viral proteins rapidly impair mitochondrial function. It is well reported that HIV‐1 proteins such as Tat and gp120 (stably expressed by the HL2/3 cell line) disrupt mitochondrial membranes, leading to loss of membrane potential and reduced ATP production (Rodríguez‐Mora et al., 2015; Shrestha et al., 2022). Except for the 48 h time point, mitochondrial activity continues to decrease steadily across the timeline. At the 96 h time point, there is a clear concentration‐dependent reduction in mitochondrial activity (*p* < 0.001).

It is important to note that the 100% supernatant treatment represents undiluted HL2/3 supernatant, which may overstate the effects of viral protein exposure. In vivo, BECs are unlikely to encounter such concentrated concentrations of viral protein; however, this model provides insight into the cellular responses in extreme conditions. This dysfunction reduces ATP production, impairs angiogenesis, and subsequently compromises BBB integrity; key events contributing to neuroinflammation and HAND (Rodríguez‐Mora et al., 2015; Shrestha et al., 2022). A critical aspect of HIV‐1 pathogenicity is to cause the host cell to remain within the second growth phase of the cell cycle and cause maximal expression of viral genes at this phase and prohibit early cell death of infected cells (Panda et al., 2023). Moreover, viral proteins, such as Tat and gp120, play a central role in this process; elucidating their impact on BECs may guide the development of therapies aimed to protect the BBB and preserve cognitive function in people living with HIV (Smail & Brew, 2018; Yang & Torbey, 2020).

### 4.3 Effect of HIV‐1 viral proteins on BBB permeability

CNS viral infections are characterized by the induced release of inflammatory cytokines by both pro‐inflammatory microglia (M1), resident in the CNS, and immune cells of the peripheral nervous system that have traversed the BBB (Mazzuca et al., 2016; Osborne et al., 2020; Strazza et al., 2011). The monopolization of the immune system by an HIV‐1 infection potentiates viral replication, especially within viral reservoirs such as the CNS (Osborne et al., 2020).

In this study, a bicameral system was employed, which provides a 2‐D construct of an in vitro BBB model. BEC monolayers were established either (i) luminally in monoculture and treated with CM via the luminal chamber (Figures 1a and 5a), which mimicked an HIV‐1 infection via the peripheral blood circulation and (ii) in co‐culture with HL2/3 cells (abluminally seeded) (Figures 1b and 6b), which mimicked a CNS infection. Thereafter, the established in vitro BBB models were exposed to HIV‐1 viral proteins to assess changes in BEC monolayer permeability.

In Figure 5, increasing concentrations of CM were experimentally analogous to increasing concentrations of HIV‐1 viral protein load in vitro, subsequently resulting in a concentration‐dependent increase in BBB permeability. The increase in permeability across the BEC monolayer is likely a resultant consequence of underlying disruption via transcellular and/or paracellular routes, the latter affecting paracellular TJs structurally, while the former affecting the functionality and/or expression of ion channels across the transmembrane domain of BECs. HIV‐1 Tat proteins are reported to cause suppressed expression in TJ claudin‐5 while also suppressing ZO‐1 (Patel et al., 2017), while Gp120 causes the breakdown of claudin‐5 and laminin, by the upregulation of MMP expression (Atluri et al., 2015; Liao et al., 2020; Louboutin et al., 2011; Patel et al., 2017).

The induction of b.End5 cell increased permeability could, therefore, have occurred through many mechanisms carried out by select HIV‐1 proteins: Gp120 envelop proteins are known for playing a key role in viral entry into host cells. It can directly interact with BECs, activating signaling pathways that lead to the production of pro‐inflammatory cytokines and chemokines (Strazza et al., 2011). In addition, both Gp120 and Vpr can cause the release of inflammatory mediators, which have been reported to disrupt TJs and increase BBB paracellular permeability, allowing immune cells and viral particles to pass through into the brain (Ronaldson & Davis, 2020). Tat proteins increased BBB permeability by inducing the expression of MMPs by BEC, contributing to the breakdown of TJs.

In the in vitro experimental context, the co‐culture of b.End5 cells with HL2/3 cells effectively represented a scenario of 100% exposure to HIV‐1 proteins, given that all HIV‐1 proteins secreted by the HL2/3 cells encountered the basolateral region of the b.End5 cell monolayer. Consequently, a comparative study involving 100% exposure to HL2/3‐CM was also conducted to assess the impact on BBB permeability in each of these conditions. The results observably indicate that b.End5 cells in the co‐culture setting exhibited a slightly greater statistical reduction in TEER (Figure 6b) when compared to b.End5 cells exposed to 100% HL2/3‐CM (Figure 6b). The discrepancy between these two exposure groups is the slightly increased permeability observed in the co‐culture systems compared to the monoculture which was treated with 100% CM is probably due to the direct effects of the HIV‐1 secreted proteins on the basolateral surface of the b.End5 monolayer, whereas the CM affects the endothelial monolayer via the luminal surface, indicating that CNS‐based HIV infection may be more detrimental than viraemic infection to brain capillary permeability. However, these two experiments: the co‐culture and the CM TEER data (Figure 6b), were closely correlated, which verified and validated the CM result seen in Figure 7b.

Our data using the in vitro bicameral BBB model strongly correlates with the in vitro data (Cooper et al., 2012; Paradis et al., 2015; Rado et al., 2022) in the literature, emphasizing the significance of this experimental design for the examination of HIV proteins and their influence on BBB permeability.

### 4.4 HIV‐1 viral protein effect on BBB morphology in a Viraemic vs. CNS viral infection

To date, HIV‐1‐induced changes in BEC morphology and topography have not yet been reported in the literature. A study by Mentor and Fisher (2021) investigated the morphological establishment of the in vitro BBB by analyzing direct intercellular communication between BECs at high resolution. The current research study further endorses the importance of cell–cell communication as a critical prerequisite for TJ localization and interaction to ensure paracellular occlusion during barrier genesis. The comparative investigation of the experimental effect of HIV systemic and CNS infection on the nano‐anatomical architecture of the b.End5 using HRSEM highlighted the ultrastructural breakdown observed within the BEC monolayer upon exposure to the HIV‐viral proteins in (Figures 7 and 8). These findings suggest a disruption in the ultrastructural integrity of the brain's endothelial barrier, which resulted in the progressive increase in permeability observed when BECs are exposed to HIV‐1 viral proteins. By juxtaposing the morphological findings with our TEER data, we can correlatively infer that a relationship exists between nano‐structural paracellular breakdown and the progressive increase in permeability (Figures 5, 6). The alignment between these two sets of data highlights the ability of HIV‐1 viral proteins to bring about misalignment of adjacent BEC membranes, which undergirds the increased permeability observed across BEC monolayers.

Our data on HIV‐viral‐protein‐induced suppressed cell division and the increased permeability was supported by the morphological findings, where (Figures 7c and 8c) denotes the disruption of the BEC monolayer and visible breakdown of direct intercellular interaction within the co‐culture system, relative to the monoculture system (Figures 7b and 8b), compared to the control conditions (Figures 7a and 8a) in which the depiction of visible BECs cell–cell interaction necessary for barrier reinforcement is observed. The degree of the formation of cell–cell interaction via TUNTs and TENTs (see Mentor & Fisher, 2021 for review) and subsequent BEC monolayer establishment directly influences the degree of permeability observed across the BEC monolayer in a viraemic scenario (CM‐treated monolayers) (Figure 5b) compared to the increased permeability seen in the context of a CNS infection (co‐culture experiments) (Figure 6b).

Based on the literature, we postulate that the permeability of b.End5 cells is considerably more affected when co‐cultured with HL2/3 cells, which alludes to the fact that a CNS HIV infection, which leads to the formation of HIV reservoirs within the CNS, has the propensity to elicit a more substantial long‐term disruption of the BBB compared to HIV infection present in the peripheral blood circulation. In an in vivo setting, microglial cells, including astrocytes, can also be susceptible to HIV infection, making them reactive (Cysique & Brew, 2019; Marincowitz et al., 2019). Given that astrocytes are integral components of the neurovascular unit, a greater disruption of the BBB in the context of CNS infection is a probable outcome (Caligaris et al., 2021).

## 5 CONCLUSION

The BBB remains a bone of contention, in the context of brain disease, and thus the amelioration of neural pathology is fraught with difficulty. To date, a balancing act persists between disease‐induced vascular “leakage” and the selectively permeable nature of the BBB, which in the context of a disease, such as an HIV‐1 infection, inhibits the successful entry of antiretroviral drugs into the brain's microenvironment.

In our model, we observe dose‐related increases in permeability. These preliminary permeability assays suggest that the magnitude of leakiness may be regulated, which would be an important consideration to guarantee effective drug delivery at therapeutic concentrations. This presents a valuable opportunity for future experiments to quantitatively assess the degree of permeability required for CNS drug delivery without inducing detrimental neuroinflammation. The findings in this study underscore novel physiological and morphological changes induced by exposing the BBB endothelium to HIV proteins. This highlights potential therapeutic and clinical amendments to help mitigate the impact of HIV‐induced inflammation on the brain microvasculature and subsequently, the CNS. In summary, the findings effectively demonstrate the indiscriminate detrimental effects that HIV‐1 proteins exert on the viability, proliferation, and mitochondrial activity of the BECs, which is the primary functional component of the BBB. Although proposed targeted follow‐up studies will aid in identifying specific HIV‐1 viral proteins as specific mediators of BBB disruption. Our study shows increased BBB permeability during apical and basolateral exposure to HIV‐1 viral proteins was endorsed by the morphological findings, which exhibit distinct structural changes in the in vitro BBB model, viz. brought about by HIV‐derived protein effects on BEC monolayers in the context of homologating a CNS infection in vitro. Exposure of the BECs to HIV proteins suppressed the formation of apicolateral NTs or the breakdown thereof within the paracellular spaces. The absence of apicolateral NTs will lead to the failure of BECs to laterally align correctly, which will result in the inevitable dysregulation of TJ interaction, leading to increased long‐term BBB permeability dysregulation which may be central to the formation of HAND in HIV patients.

## 6 FUTURE PERSPECTIVES

Research exploring HIV‐1 viral protein‐induced alteration in the structural integrity of the BBB will make any uncertainties regarding the relationship between barrier integrity and physical interruption of barrier genesis, ensued by viral infection, less obfuscate. Illuminating the role of ultrastructures in the reinforcement of the protective properties of the endothelial barrier and bringing to the fore subsequent detrimental effects on morphology caused by HIV assists in gauging the long‐term effects of the viral proteins on the cerebral microvasculature. Furthermore, introducing combination anti‐retroviral therapy (cART) for HIV‐1 infection on cerebrovascular disease by exploring underpinning mechanistic effects both in vitro and incorporating *in vivo* studies will expand on these findings and will broaden the landscape for studying direct cell–cell communication and/or miscommunication in the context of HIV‐associated neuroinflammation and the long‐term toxic effects of cART on the architecture of the brain's protective barrier.

## FUNDING INFORMATION

This research was supported by the Start‐up Emerging Researcher Award (UCT), which facilitated the initial stages of this project. Additionally, the study received funding from the National Research Foundation (NRF)–Thuthuka Grant [TTK230509103990]. The views expressed in this publication are those of the authors and do not necessarily reflect the views of the NRF.

## CONFLICT OF INTEREST STATEMENT

The authors disclose that there is no conflict of interest concerning the publishing of this manuscript.

## Data Availability

All relevant experimental data is archived within Stellenbosch University (SU) archives and is available as per SU data and intellectual property policy guidelines and its associated copyright protection. The data that support the findings of this study are available from the corresponding author upon reasonable request.
